# Structure, morphology, optical properties, and Judd–Ofelt analysis of YP_(1−*x*)_V_*x*_O_4_:Eu^3+^ materials synthesized by the combustion method

**DOI:** 10.1039/d4na01052c

**Published:** 2025-05-27

**Authors:** Nguyen Vu, Ngo Khac Khong Minh, Thai Thi Dieu Hien, Pham Duc Roan, Lam Thi Kieu Giang, Nguyen Thanh Huong, Hoang Thi Khuyen, Pham Thi Lien, Dinh Manh Tien, Nguyen Trung Kien, Dao Ngoc Nhiem

**Affiliations:** a Institute of Materials Sciences, Vietnam Academy of Science and Technology 18 Hoang Quoc Viet Street Hanoi 100000 Vietnam nguyenvu@ims.vast.ac.vn ntkien@ims.vast.ac.vn; b Can Tho University of Technology 256 Nguyen Van Cu Street Can Tho City 900000 Vietnam; c Faculty of Chemistry, Hanoi National University of Education 136 Xuan Thuy Street Hanoi 100000 Vietnam

## Abstract

YP_(1−*x*)_V_*x*_O_4_:Eu^3+^ materials were synthesized *via* a simple combustion method. Material characterization illustrated the formation of spherical particles with a tetragonal crystal structure and a uniform size of 20 nm, although aggregation was observed. Fluorescence spectroscopy was then employed to explore the optical characteristics, revealing key insights into the luminescent behavior of the as-prepared materials. A detailed examination of the branching ratio of the ^5^D_0_ → ^7^F_2_ electronic transition relative to the ^5^D_0_ → ^7^F_1_ transition was performed, which is closely tied to the symmetry of the local environment of the Eu^3+^ activators. This investigation utilized Judd–Ofelt theory to calculate intensity and emission parameters. Additionally, the fluorescence lifetime of the material was measured under various V/P ratios, elucidating the relationship between these variables. Finally, the emission color and correlated color temperature (CCT) of the synthesized material were evaluated through the CIE 1931 chromaticity diagram, confirming its potential for use in optical applications based on its tunable emission characteristics.

## Abstract 2

Spherical YP_1−*x*_V_*x*_O_4_:5% Eu^3+^ nanoparticles prepared by a simple combustion method exhibit a long lifetime red emission under UV irradiation exposure.

## Graphical abstract

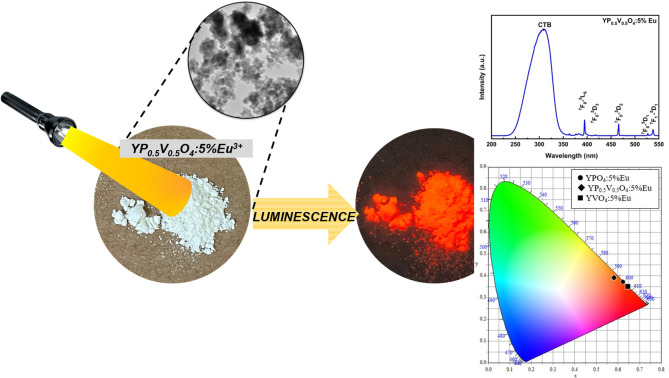

## 1. Introduction

Luminescence, the emission of light by specific materials at relatively low temperatures, has hooked numerous material researchers and has become an important aspect of modern nanomaterial science and technology since the last century. Luminescence is referred to as cold light to distinguish it from incandescence (hot light) like firelight, candlelight, or other modern incandescent lamps of the twentieth century.^1^ Lanthanide or rare-earth ion doped luminescent nanomaterials are indeed a significant research area due to their unique ability to emit strong fluorescence through either 5d–4f transitions or faint 4f–4f transitions when excited using various irradiation sources with exceptional photostability and long luminescence lifetimes.^1,2^ The fact is that, at the moment, lanthanide luminescence plays a crucial role in multiple applications, including lighting, telecommunications, solar energy conversion, lasers, luminescent molecular thermometers, and others.^2–5^

The luminescence efficiency and the resultant color are influenced by several factors. The host material in which the rare earth ions are embedded plays a crucial role in determining the overall luminescent properties. Different host matrices can affect the energy transfer processes and the stability of the luminescent centers.^6,7^ Moreover, the activator ions, commonly rare earth ions such as europium (Eu), are responsible for the emission of light.^8^ The concentration of these ions must be optimized to maximize the luminescence efficiency while avoiding quenching effects that can occur at higher concentrations.^9^ In addition, the synthesis conditions, including temperature and method, such as solid-state reaction, sol–gel process, and hydrothermal synthesis, significantly influence the crystallinity, particle size, and homogeneity of the luminescent material.^10–12^

Among lanthanide luminescent nanomaterials, phosphates and vanadates, such as YPO_4_:Eu^3+^ and YVO_4_:Eu^3+^, are notable for their ability to emit light in the orange-red to pure red spectrum.^13,14^ This characteristic emission makes them highly suitable for applications in fluorescent lamps and emissive displays.^15,16^ However, the emission properties of these materials are primarily due to the Eu^3+^ ions, which serve as activators. When mixing the host lattices of YPO_4_ and YVO_4_, one can achieve the desired luminescence. The color of the emitted light can be fine-tuned from orange-red to pure red by partially replacing VO_4_^3−^ groups with PO_4_^3−^ groups in the host lattice.^17,18^ This substitution affects the local environment of the Eu^3+^ ions, thereby altering the energy levels and the resultant emission spectrum. This study focuses on Y(PO_4_)_*x*_(VO_4_)_(1−*x*)_, a phosphor material, to analyze how varying the ratio of phosphate (PO_4_^3−^) to vanadate (VO_4_^3−^) groups affects its optical properties. These materials were synthesized using the combustion method, a technique known for producing high-purity and homogeneously distributed phosphor powders.^19^ The study delves into understanding the local environment around Eu^3+^ ions since the symmetry and bonding nature of the surrounding ligands significantly influence the Eu^3+^ emission characteristics. Judd–Ofelt theory is applied to analyze these effects in detail.

## 2. Experimental

### 2.1. Chemicals

All reagents, including Y_2_O_3_, Eu_2_O_3_, HNO_3_, NH_3_ solution, H_3_PO_4_, NH_4_VO_3_, and analytical grade urea, were purchased from Merck and used directly without any further purification.

### 2.2. Materials preparation

For the synthesis of YP_1*-x*_V_*x*_O_4_, Y_2_O_3_ and Eu_2_O_3_ precursors were first dissolved in concentrated nitric acid (HNO_3_) to form Y(NO_3_)_3_ and Eu(NO_3_)_3_ respectively. After that, the Y(NO_3_)_3_ and Eu(NO_3_)_3_ solutions with the desired molar ratios were mixed in another beaker placed on a magnetic stirrer and then diluted later with distilled water. Subsequently, urea as a fuel was then added to the mixtures and stirred continuously until obtaining a clear and colorless solution (solution A). Separately, NH_4_VO_3_ was fully dissolved in distilled water at 70 °C to create a transparent yellow solution (solution B), whereas solution C was also prepared by mixing H_3_PO_4_ and NH_3_ solution with a molar ratio of 1 : 2. In the following step, solution B was gently added to solution A with continuous stirring for 30 minutes, before solution C was slowly added with vigorous stirring over 60 minutes. The resulting mixture was evaporated to produce a yellow gel-like precursor. Finally, the precursor was heated to 900 °C at a rate of 10 °C min^−1^ to obtain the desired products. The corresponding YPO_4_:5%Eu and YVO_4_:5%Eu samples for comparison were prepared through a similar procedure without the addition of the as-prepared solution B and solution C, respectively.

### 2.3. Materials characterization

The crystalline structure of the materials was analyzed using a Bruker D8 Advance X-ray diffractometer under Cu Kα radiation (*λ* = 1.5406 Å). The diffraction patterns were recorded within a 2*θ* range of 20° to 80° at a scanning speed of 0.03° s^−1^. To study the morphology of the final product, SEM was performed using a Hitachi S-4800 device (Japan). The functional groups present in the obtained materials were examined by the Fourier Transform Infrared (FTIR) technique using a Nicolet iS10 FTIR spectrometer (Thermo Scientific, USA). The examined samples were homogeneously mixed with KBr powder and pelletized for measurement, and the results were consequently corrected using the KBr background as a reference. The included elements in the obtained samples were characterized by energy-dispersive X-ray (EDX) spectroscopy using a JEOL JSM 6500F equipment (Japan). Finally, fluorescence excitation spectrum and fluorescence spectrum measurements were carried out at selected excitation wavelengths using a Horiba Fluorolog-3 device (Japan) to investigate the optical properties of Y(PO_4_)_*x*_(VO_4_)_(1−*x*)_ samples.

## 3. Results and discussion

### 3.1. Structure, morphology, and elemental analysis

**Fig. 1:**
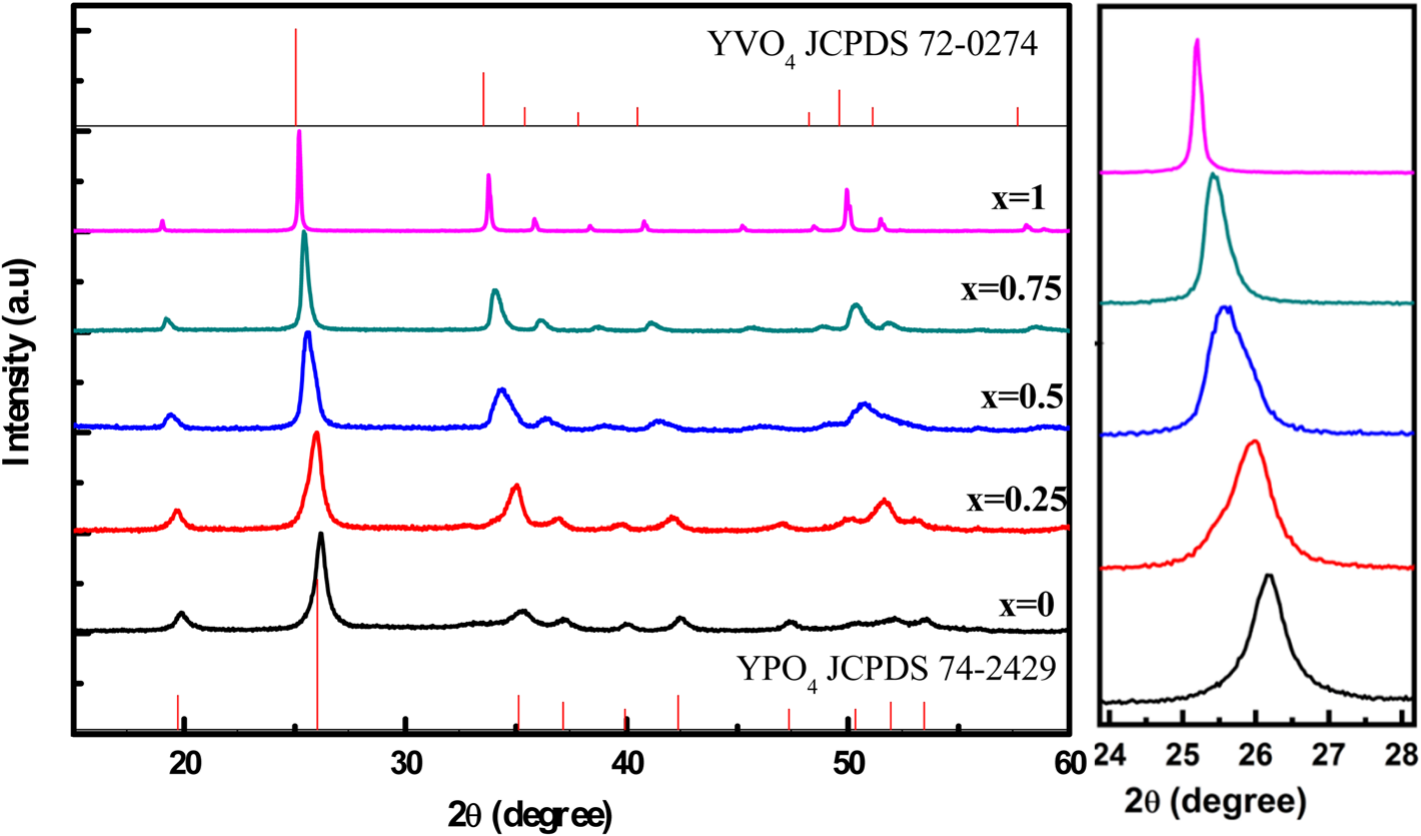
XRD patterns of YP_(1−*x*)_V_*x*_O_4_:5% Eu materials (*x* = 0, 0.25, 0.50, 0.75, and 1).

Fig. 1 shows the diffraction pattern of the Y(P_1−*x*_V_*x*_)O_4_:5% Eu material for varying compositions of vanadium (*x* = 0, 0.25, 0.50, 0.75, and 1). All diffraction peaks for the YPO_4_ sample align with the standard reference JCPDS 74-2429, confirming a single-phase material with a tetragonal crystal structure.^20^ The lattice parameters are determined to be *a* = 6.878 Å, *b* = 6.878 Å, and *c* = 6.036 Å, indicating no secondary phases or impurities due to doping. Similarly, the YVO_4_ sample exhibits diffraction peaks consistent with the standard reference JCPDS 72-0274 for YVO_4_ crystals.^21^ This sample also forms a single-phase material with a tetragonal crystal structure and lattice parameters of *a* = 7.100 Å, *b* = 7.100 Å, and *c* = 6.270 Å. The results demonstrate that both YPO_4_ and YVO_4_ materials maintain their respective crystal structures despite variations in vanadium concentration, confirming the stability of the tetragonal phase across the composition range studied.

The X-ray diffraction pattern reveals that substituting P^5+^ with V^5+^ in the YP_(1−*x*)_V_*x*_O_4_:5% Eu lattice results in measurable lattice expansion, as indicated by XRD peak shifts to lower 2*θ* values. Additionally, the diffraction peaks become narrower, and the half-width decreases. This is due to the larger ionic radius of V^5+^ (0.54 Å) compared to P^5+^ (0.38 Å) in six-fold coordination. Consequently, replacing P with V results in an expansion of the lattice structure, leading to an increase in particle size. Using the Scherrer formula, the calculated particle sizes for the YP_(1−*x*)_V_*x*_O_4_:5% Eu samples are 11.2 nm (*x* = 0), 11.5 nm (*x* = 0.25), 12.3 nm (*x* = 0.5), 17.9 nm (*x* = 0.75), and 21.3 nm (*x* = 1), respectively. This gradual increase in size is consistent with the larger unit cell volume of YVO_4_ compared to YPO_4_ (a difference of approximately 10%), which aligns with the ionic radii disparity between V^5+^ and P^5+^ and further supports the structural changes caused by the substitution of vanadium for phosphorus in the crystal lattice.

**Fig. 2:**
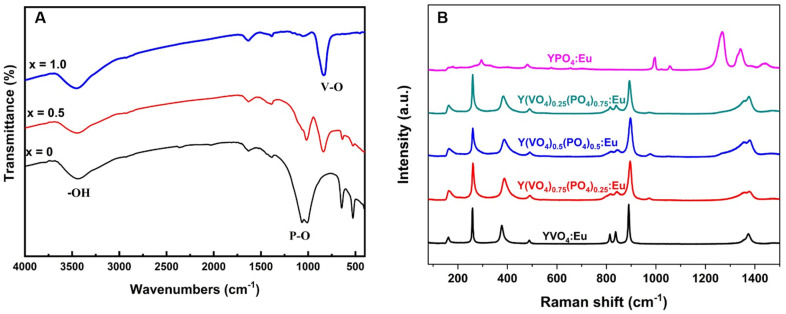
(A) FTIR and (B) Raman spectra of YP_(1−*x*)_V_*x*_O_4_:5% Eu materials.

In addition to the XRD technique, the FTIR spectra provide further insight into the phase formation of YP_(1−*x*)_V_*x*_O_4_:Eu materials. Fig. 2 shows the IR spectra of YPO_4_:5% Eu, Y(P_0.5_V_0.5_)O_4_:5% Eu, and YVO_4_:5% Eu. All the spectra exhibit signals in the range of 3500–3450 cm^−1^ and 1640–1615 cm^−1^, which correspond to the stretching and bending vibrations of the OH group in water molecules.^22^ The absorption band at 1043–1084 cm^−1^ is attributed to the stretching vibrations of the P–O bond in the phosphate group, indicating the presence of phosphorus in the lattice.^23^ Additionally, the peaks observed at 824 cm^−1^ and 453 cm^−1^ are associated with the vibrations of the V–O bond in the vanadate group and the Eu–O bond, respectively.^24^ These distinctive absorption peaks confirm the successful incorporation of vanadium and europium into the crystal structure, reflecting changes in the chemical bonding environment due to varying V/P ratios. The IR spectra, in conjunction with the X-ray diffraction data, validate the phase stability and formation of YP_(1−*x*)_V_*x*_O_4_:Eu materials with consistent structural characteristics.

A subsequent study using Raman spectroscopy was performed to support the results of XRD and FTIR techniques (Fig. 2B). The vibration modes of both YPO_4_:Eu^3+^ and YVO_4_:Eu^3+^ were in agreement with previously reported data by Yahiaoui *et al.*^25^ and Mitrić *et al.*^26^ In the case of YVO_4_:Eu^3+^, several sharp Raman lines are observed, corresponding to the internal vibrations of the (VO_4_)^3−^ group and the external vibrations of (VO_4_)^3−^ tetrahedra and Y^3+^ ions within the YVO_4_ unit cell. Notably, the external mode E_g2_(2) at 161 cm^−1^ is attributed to the O–Y–O bending mode. The internal vibrations related to the O–V–O bending and (VO_4_)^3−^ stretching modes occur at higher frequencies: 259 (B_2g_), 337 (A_1g_(1)), 488 (B_1g_(3)), 814 (B_1g_(4)), 837 (E_g_(5)) and 890 cm^−1^ (A_1g_(2)). The spectrum is primarily dominated by the totally symmetrical vibrations of the (VO_4_)^3−^ tetrahedron. The narrow width of the Raman lines suggests that the synthesized powder has good crystallinity and homogeneity. As the content of vanadate decreases, all modes exhibit slight shifts and become broader, confirming the formation of the solid state of YP_(1−*x*)_V_*x*_O_4_:5% Eu.^27^ For YPO_4_:5% Eu, four (2B_1g_ + 2E_g_) are classified as external modes due to the translations of the (PO_4_)^3−^ and Y^3+^ ions, while one mode (E_g_) corresponds to the librational motion of the entire (PO_4_)^3−^ tetrahedral. Additionally, seven internal modes (2A_1g_ + 2B_1g_ + 1B_2g_ + 2E_g_) arise from the internal vibrations of the oxygen atoms in the (PO_4_)^3−^ tetrahedral groups.^28^ It is important to note that the validity of the Raman spectrum is above 160 cm^−1^.

**Fig. 3:**
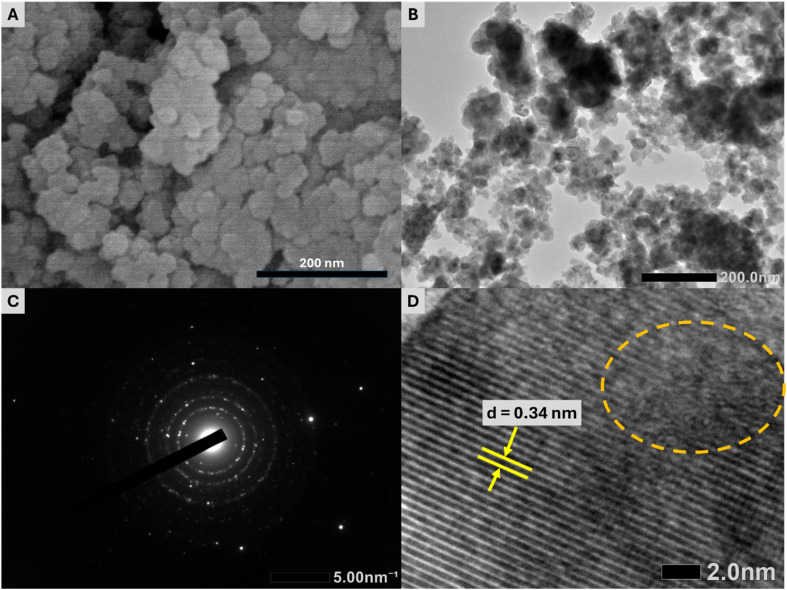
(A) SEM, (B) TEM, (C) SAED, and (D) HR-TEM images of the Y(P_0.5_V_0.5_)O_4_:5% Eu sample.

**Fig. 4:**
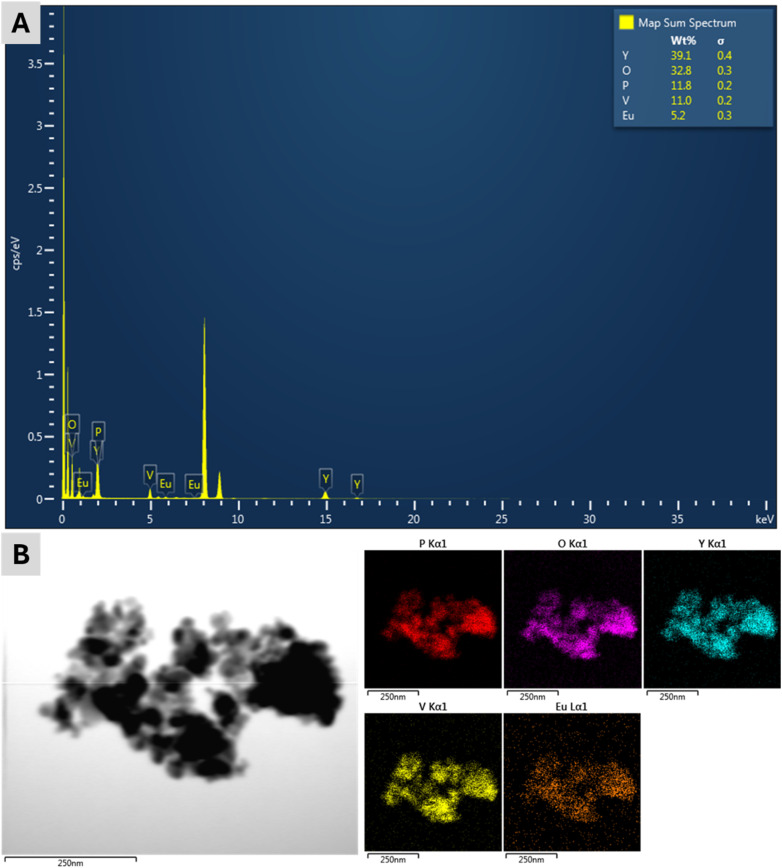
(A) EDX spectra and (B) mapping of elements in the Y(P_0.5_V_0.5_)O_4_:5% Eu sample.

The SEM and TEM images reveal the spherical shape and the uniformity in size of the synthesized Y(P_0.5_V_0.5_)O_4_:5% Eu sample (Fig. 3A and B). The average grain size is approximately 20 nm, which is in good agreement with the particle size theoretically derived from the XRD results. This consistency of SEM, TEM and XRD results further validates the successful synthesis of a homogeneous and well-defined nanostructure of Y(P_0.5_V_0.5_)O_4_:5% Eu samples as well as suggests a controlled crystal growth process. Nonetheless, in the SEM and TEM images, the aggregation of the examined particles can also be observed. In this study, the synthesized particles were in the nanoscale range (∼20 nm) and showed some aggregation; however, no measures were reported to ensure monodispersity. For applications like micro-LEDs, where uniform optical properties are crucial, a narrow size distribution is essential. Future work will address this by using surfactants or capping agents during synthesis to control particle growth or applying post-synthesis techniques such as size-selective precipitation, centrifugation, or surface functionalization to reduce aggregation and improve dispersion. On the other side, a captured SAED pattern indicates the polycrystalline nature of the Y(P_0.5_V_0.5_)O_4_:5% Eu sample (Fig. 3C), whereas the HR-TEM image shows a fringe spacing of 0.34 nm, which corresponds to the fringe spacing of the (200) planes (Fig. 3D). The formation of defects on the surface of the Y(P_0.5_V_0.5_)O_4_:5% Eu sample was also detected and marked by a dashed circle in the HR-TEM image. Furthermore, EDX spectra and elemental mapping confirm the presence and distribution of Y, P, V, O, and Eu in the Y(P_0.5_V_0.5_)O_4_:5% Eu sample without the existence of other impurities (Fig. 4).

### 3.2. Optical properties

**Fig. 5:**
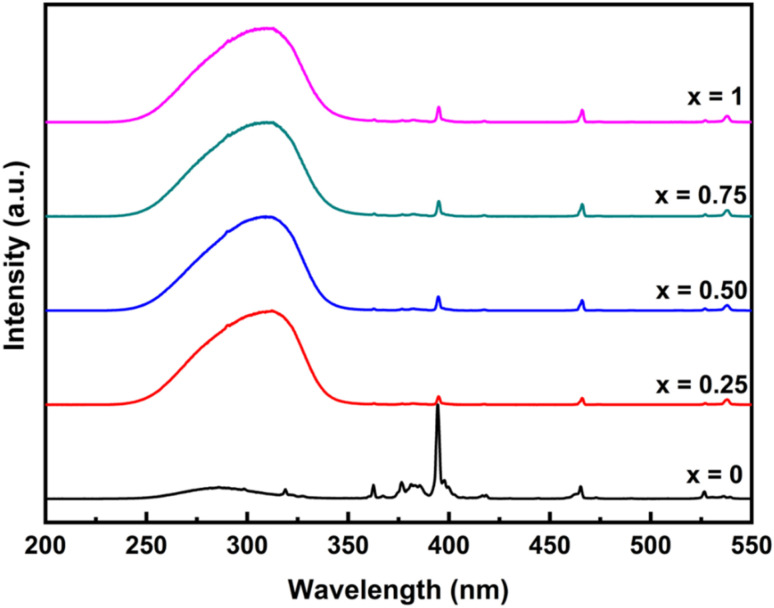
Fluorescence excitation spectrum of Y(P_1−*x*_V_*x*_)O_4_:5% Eu materials (*x* = 0, 0.25, 0.5, 0.75, and 1).

The optical properties of YP_(1−*x*)_V_*x*_O_4_:5% Eu materials were analyzed using fluorescence spectroscopy to explore the effects of varying the P/V ratio on their luminescence behavior. The fluorescence excitation spectrum for the emission at 619 nm (Fig. 5) features a broad band along with several sharp, narrow peaks. The broadband, spanning the wavelength range of 200–350 nm, corresponds to the lattice excitation and the charge transfer band (CTB). For YPO_4_:5% Eu, the broad band observed around 250–300 nm is attributed to the charge transfer transition between oxygen and europium.^29^ This occurs due to the movement of electrons from the 2p orbital of oxygen to the empty 4f orbital of Eu^3+^. In the Y(P_1−*x*_V_*x*_)O_4_:5% Eu samples (for *x* ≠ 0), as the concentration of vanadium increases, the intensity of this excitation absorption band becomes more pronounced compared to that of YPO_4_. Additionally, the excitation absorption peak shifts toward longer wavelengths. This redshift is due to the presence of a new absorption band around 315 nm, corresponding to the vanadate group.^30^ This band is linked to the charge transfer process between oxygen and vanadium, where electrons transition from the 2p orbital of oxygen to the 3d orbital of vanadium.

**Fig. 6:**
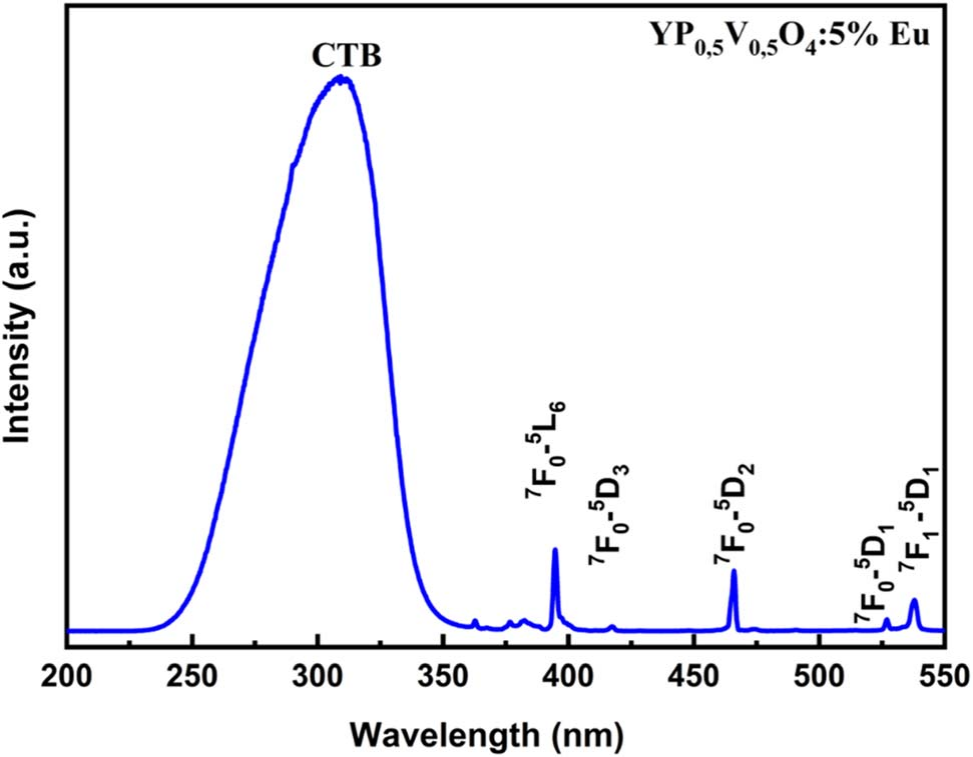
The fluorescence excitation spectrum of the Y(P_0.5_V_0.5_)O_4_:5% Eu material.

In the fluorescence excitation spectrum shown in Fig. 6, several sharp and narrow lines are observed in the 350–550 nm range. These lines correspond to the characteristic electron transitions of Eu^3+^ ions, where the host lattice does not participate in the absorption process. Specifically, these transitions are associated with the following energy levels of Eu^3+^: ^7^F_0_ → ^5^D_4_, ^7^F_0_ → ^5^G_4_, ^7^F_0_ → ^5^L_6_, ^7^F_0_ → ^5^D_3_, ^7^F_0_ → ^5^D_2_, ^7^F_0_ → ^5^D_1_, ^7^F_0_ → ^5^D_1_ corresponding to the transition wavelengths 362, 376, 384, 395, 418, 466, 526, and 538 nm, respectively.^22,25,29^ These sharp lines indicate direct f–f transitions within the Eu^3+^ ion, reflecting its electronic configuration and energy level splitting. In addition, it can be observed that the intense charge transfer (CT) band around 315 nm was stronger than the sharp f–f excitation line at 395 nm. This contrasts with typical Eu^3+^-doped systems, where f–f transitions usually dominate due to weak CT absorption. The enhanced CTB in this material likely stems from the high covalency of the Eu–O bond in the phosphate–vanadate matrix, which increases O^2−^→Eu^3+^ CT probability. Additionally, the mixed-ligand environment (PO_4_^3−^ and VO_4_^3−^) may distort the local symmetry around Eu^3+^ ions, relaxing selection rules and boosting CT absorption.

**Fig. 7:**
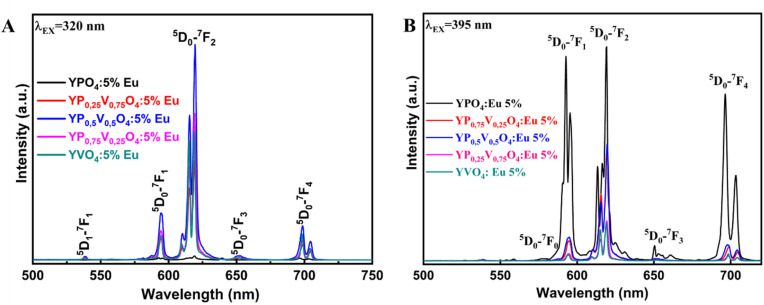
Fluorescence spectra of Y(P_1−*x*_V_*x*_)O_4_:5% Eu samples under (A) 320 nm and (B) 395 nm excitation.

The fluorescence spectrum of the YP_(1−*x*)_V_*x*_O_4_:5% Eu material under 320 nm excitation (Fig. 7A) reveals characteristic transitions of Eu^3+^ ions as vanadium (V) replaces phosphorus (P) at *x* = 0, 0.25, 0.5, 0.75, and 1, respectively. Specifically, the spectra show emission peaks corresponding to the following Eu^3+^ transitions at 594 nm (^5^D_0_ → ^7^F_1_), 614–620 nm (^5^D_0_ → ^7^F_2_), 650 nm (^5^D_0_ → ^7^F_3_) and 696–703 nm (^5^D_0_ → ^7^F_4_). As the ratio of V increases, the intensity of the ^5^D_0_–^7^F_2_ transition becomes more dominant compared to the other emissions.^22,29,31^ This indicates that the Eu^3+^ ions are occupying positions within the crystal lattice that lack an inversion center of symmetry. Let *R* be the asymmetry ratio given by:1
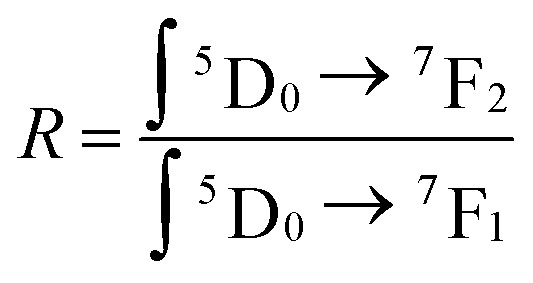


The higher the *R* coefficient, the greater the emission (^5^D_0_ → ^7^F_2_) and the stronger the red application of the material, opening the prospect of exploiting the red application of the material.

The results of the experimental branching ratio of the ^5^D_0_ → ^7^F_2_ transition and the emission asymmetry ratio are presented in Table 1.

**Table 1:** Optical parameters of Y(P_1−*x*_V_*x*_)O_4_:5% Eu samples under different excitation wavelengths

Samples	Excitation wavelength		Excitation wavelength	
	320 nm		395 nm	
	Transfer branching ratio ^5^D_0_–^7^F_2_	Asymmetry ratio	Transfer branching ratio ^5^D_0_–^7^F_2_	Asymmetry ratio
YPO_4_:5% Eu	58.34%	2.91	29.10%	0.92
Y(P_0.75_V_0.25_)O_4_:5% Eu	64.04%	4.61	57.73%	3.07
Y(P_0.5_V_0.5_)O_4_:5% Eu	66.35%	4.76	66.49%	4.89
Y(P_0.25_V_0.75_)O_4_:5% Eu	67.12%	5.6	67.87%	5.99
YVO_4_:5% Eu	68.03%	5.94	68.29%	4.51

The fluorescence spectrum of Y(P_1−*x*_V_*x*_)O_4_:5% Eu under 395 nm excitation (Fig. 7B) reveals characteristic Eu^3+^ transitions in all material samples. Notably, YPO_4_:5% Eu demonstrates the highest emission intensity compared to other compositions, making it the most optimal choice for strong fluorescence under 395 nm excitation. Additionally, the emission intensities vary with different substitution ratios of V for P, indicating that even small compositional changes can influence fluorescence properties. This variation is captured in the optical parameters of Y(P_1−*x*_V_*x*_)O_4_:5% Eu under 395 nm excitation (Table 1). Therefore, the choice of excitation wavelength significantly impacts the fluorescence intensity of these materials. Depending on the desired emission characteristics (red, orange, or red-orange), selecting the right excitation wavelength is crucial for tailoring the optical performance of these materials.

### 3.3. Judd–Ofelt theory for Eu^3+^ ions

Judd–Ofelt (JO) theory is a widely adopted method for analyzing the optical properties of rare-earth (RE^3+^) ions in solid-state materials. By estimating the intensity parameters *Ω*_*λ*_ (*λ* = 2, 4, 6), JO theory provides insights into the ligand field environment and radiative properties of the doped ions. The standard application of JO theory typically relies on absorption spectra to calculate these parameters. However, applying this technique to Eu^3+^ ions presents challenges due to their inherently weak absorption bands, making accurate determination of the intensity parameters difficult. To address this limitation, Krupke introduced an alternative approach for calculating the *Ω*_*λ*_ parameters using the emission spectrum of Eu^3+^ ions. This method leverages the higher sensitivity of emission spectra, which allows for a more precise determination of the intensity parameters. By utilizing the emission data, Krupke's method offers a practical solution for evaluating the optical properties of Eu^3+^-doped compounds.

According to this method, the intensity parameters *Ω*_*λ*_ are derived using the emission spectrum. Specifically, the method utilizes the magnetic dipole (MD) transition ^5^D_0_ → ^7^F_1_ and the electric dipole (ED) transitions ^5^D_0_ → ^7^F_2,4,6_. The transition probability A_MD_ of the ^5^D_0_ → ^7^F_1_ allowed MD transition can be calculated using the following expression^32^ (eqn (2))2
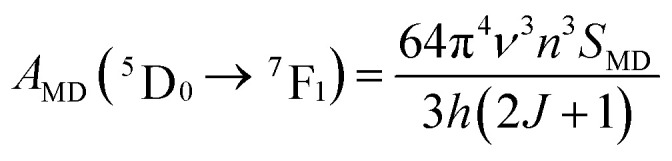


The total angular momentum *J* of the ^5^D_0_ state is *J* = 0, *ν* is the energy of the ^5^D_0_ → ^7^F_1_ transition, and *n* is the refractive index of the host matrix (*n* = 1.945 (YVO_4_) and *n* = 1.732 (YPO_4_)). The parameter *S*_MD_ = 9.6 × 10^−42^ (esu^2^ cm^2^) is a constant for a given rare-earth ion, reflecting the magnetic dipole nature of the transition, and it typically does not vary significantly between different host matrices. Thus, the *A*_MD_ (^5^D_0_ → ^7^F_1_) parameter can be calculated using the equation^32^ (eqn (3))3
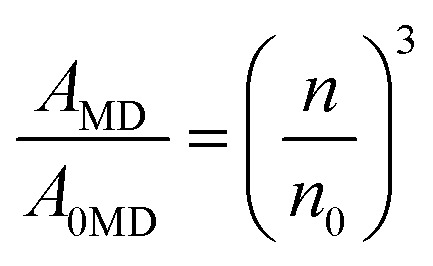
where *A*_0MD_ and *n*_0_ are the transition probability and refractive index for a reference material (from published data), respectively. The transition probability for the allowed electric dipole (ED) transitions, such as ^5^D_0_ → ^7^F_2,4,6_, can be calculated using the following formula^32^ (eqn (4))4
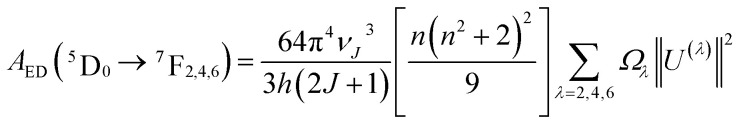
where ‖*U*^(*λ*)^‖^2^ (*λ* = 2, 4, 6) are the squared doubly reduced matrix elements for the ^5^D_0_ → ^7^F_*J*_ (*J* = 2, 4, 6) transitions. By divided eqn (4) by eqn (2), eqn (5) is obtained as follows:^32^5
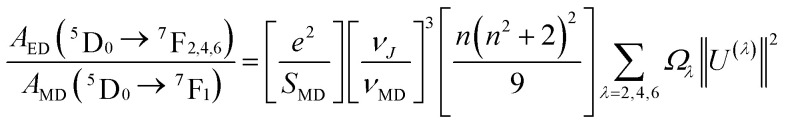


For all electric dipole (ED) transitions originating from the ^5^D_0_ level, the reduced matrix elements are zero except for the ^5^D_0_ → ^7^F_2_ (*U*^(2)^ = 0.0032), ^5^D_0_ → ^7^F_4_ (*U*^(4)^ = 0.0023) and ^5^D_0_ → ^7^F_6_ (*U*^(6)^ = 0.0002) transitions. The ^5^D_0_ → ^7^F_*J*_ (*J* = 1, 2, 4, 6) transitions are used for the determination of the radiative transition probabilities, while ^5^D_0_ → ^7^F_*J*_ (*J* = 0, 3, 5) transitions are prohibited and are not included in JO calculation. The ^5^D_0_ → ^7^F_6_ transition related to the *U*^(2)^ parameter was not included in the calculation because it could not be detected by PL in the infrared region.

**Fig. 8:**
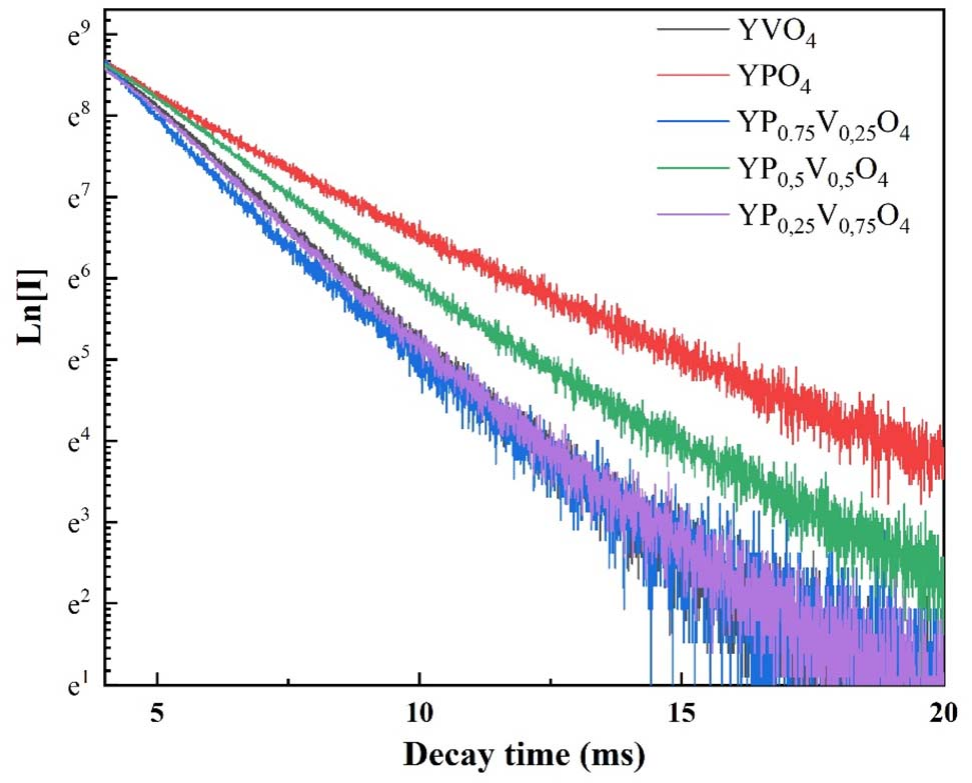
Luminescence decay curves for Y(P_1−*x*_V_*x*_)O_4_:5% Eu.

Using the emission spectrum in Fig. 8, eqn (5), and parameters *U*^(*λ*)^, the intensity parameters (*Ω*_2,4_) were calculated for all samples and are shown in Table 2.

**Table 2:** The *R* ratio and *Ω*_2,4_ intensity parameters for Y(P_1−*x*_V_*x*_)O_4_:5% Eu

Samples	*R*	*Ω*_2_ ×10^−20^	*Ω*_4_ ×10^−20^	*Ω*_6_ ×10^−20^	Ref.
		cm^2^	cm^2^	cm^2^	
YPO_4_:5% Eu	1.14	1.74	2.32	0	—
YP_0.75_V_0.25_O_4_:5% Eu	3.30	5.34	3.08	0	—
YP_0.5_V_0.5_O_4_:5% Eu	5.82	9.32	3.30	0	—
YP_0.25_V_0.75_O_4_:5% Eu	6.67	10.83	3.07	0	—
YVO_4_:5% Eu	7.21	11.73	2.79	0	—
Gd_2_O_3_:5% Eu		4.46	0.73	0	29
LaF_3_:Eu^3+^	—	1.19	1.16	0.39	33

It is known that the *Ω*_2_ parameter is indeed sensitive to the asymmetry of the local environment and the covalent nature of the Eu^3+^–ligand bond. When *Ω*_2_ increases, it often indicates greater asymmetry and stronger covalent bonding between the Eu^3+^ ions and their surrounding ligands. On the other hand, *Ω*_4_ is largely dependent on the rigidity and structural properties of the host material. An increase in the *Ω*_4_ parameter indicates that the environment surrounding the Eu^3+^ ions has reduced rigidity and becomes more flexible. The results presented in Table 2 indicate that both the *R*-value and *Ω*_2_ parameters increase as the V/P ratio rises. This trend suggests that the optical properties of the europium (Eu^3+^) doped material are significantly influenced by the change in the volume-to-phosphorus ratio. An increase in the *R*-value means that the ^5^D_0_ → ^7^F_2_ transition, corresponding to the red emission peak, becomes more pronounced compared to the ^5^D_0_ → ^7^F_1_ transition. This suggests that the material produces a purer and more intense red emission as the V/P ratio increases, which could be attributed to a change in the local environment of the Eu^3+^ ions. An increase in *Ω*_2_ indicates that the Eu^3+^ ions are situated in a more asymmetric environment, which could result from changes in the material's structure or composition as the V/P ratio increases. Moreover, the increase in *Ω*_2_ suggests a higher degree of covalency in the Eu–ligand bonds, which enhances the electric dipole transition probability, making the ^5^D_0_ → ^7^F_2_ transition more intense. In particular, when the V/P ratio exceeds 1, the *Ω*_2_ parameter reaches a significantly higher value compared to the *Ω*_2_ values typically observed in standard materials such as Gd_2_O_3_:5% Eu and LaF_3_:Eu^3+^.

In the study of YPVO_4_, the ^5^D_0_ → ^7^F_2_ transition at 615 nm is an electric dipole transition that becomes more dominant in intensity as vanadium (V) is gradually replaced by phosphorus (P). This replacement likely influences the crystal field environment around the Eu^3+^ ions, enhancing the asymmetry of the local site.

Branching ratios (*β*) are crucial in understanding the distribution of emission intensity among different transitions. The good agreement between calculated (*β*_cal_) and experimental (*β*_exp_) values (Table 2) highlights the accuracy of the theoretical models, likely based on Judd–Ofelt (JO) theory. The ^5^D_0_ → ^7^F_2_ transition shows a branching ratio above 60%, indicating strong electric dipole character and favorable laser transition properties. This high ratio suggests that Eu^3+^ ions occupy low-symmetry sites, which enhances radiative transition probabilities – key for efficient optical amplification in laser applications. Using JO theory, one can calculate the radiative lifetime *τ*_R_. This lifetime is an essential parameter as it provides insights into the efficiency of the luminescent process. The formula typically used is as follows (eqn (6))^32^6
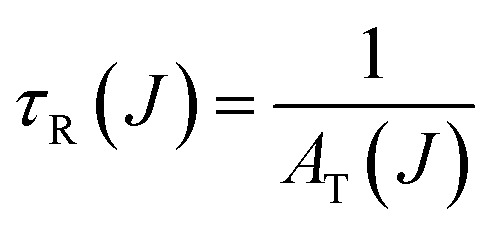
where *A*_*T*_(*J*) represents the radiative transition probabilities for transitions from ^5^D_0_ to various ^7^F_*J*_ levels.

To check the accuracy of the calculations, we measured the fluorescence decay curve (Fig. 8). The experimental setup involved exciting the sample with a 252 nm UV source, followed by monitoring the emission at 615 nm. The average lifetime was calculated using the formula (eqn (7))^19^7
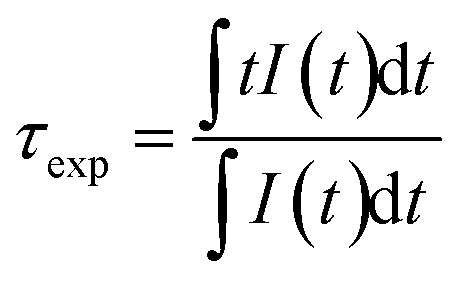


The fluorescence lifetimes of the samples are presented in Table 3. The quantum efficiency (*η*) is an important parameter in phosphor performance and can be calculated using eqn (8):^32^8
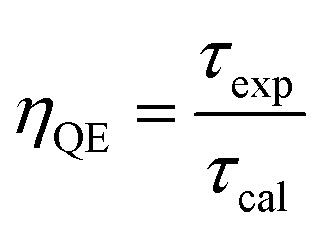
where *τ*_cal_ is the radiation lifetime determined by the Judd–Ofelt parameterization in the study, and *τ*_exp_ is the experimental fluorescence lifetime. The *η* results are summarized in Table 3, where the quantum efficiency of the phosphor increased from 45.68% to 80.89% with increasing V concentration.

**Table 3:** Lifetime (*τ*), quantum efficiency (*η*), and branching ratios of Y(P_1−*x*_V_*x*_)O_4_:5% Eu

Samples	*β*_exp_ (%)	*β*_cal_ (%)	*τ*_R_ (ms)	*τ*_exp_ (ms)	*η* (%)
YPO_4_:5% Eu	44.67	45.61	4.75	2.17	45.68
YP_0.75_V_0.25_O_4_:5% Eu	61.67	63.06	3.90	1.58	40.51
YP_0.5_V_0.5_O_4_:5% Eu	72.78	74.23	2.63	2.16	82.13
YP_0.25_V_0.75_O_4_:5% Eu	73.53	77.65	2.37	1.72	72.57
YVO_4_:5% Eu	75.08	79.81	2.25	1.82	80.89

**Fig. 9:**
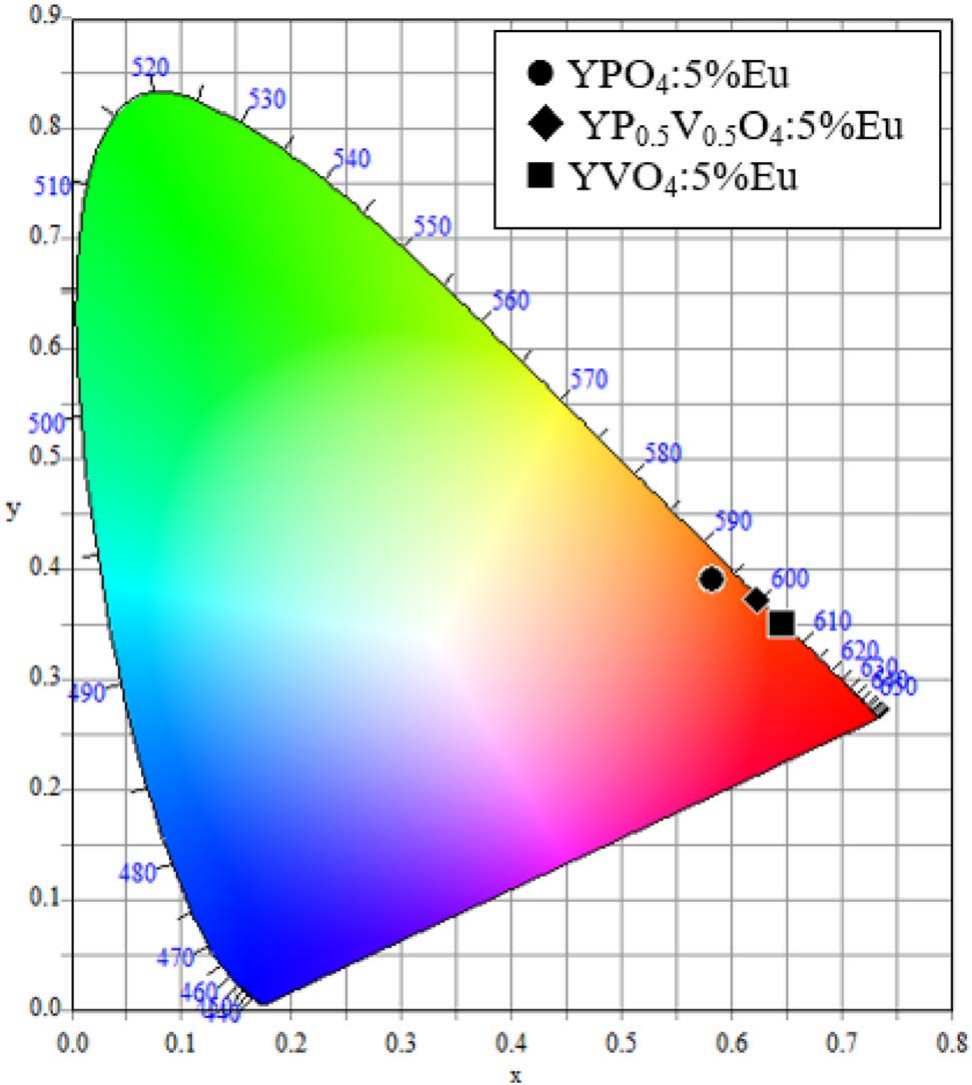
CIE chromaticity diagram for emission spectra of YP_(1−*x*)_V_*x*_O_4_:5% Eu^3+^ (*x* = 0, 0.5, 1).

The CIE chromaticity diagram was used to illustrate the color of the recorded luminescence. As shown in Fig. 9, the calculated *x* and *y* coordinates are positioned within the red region. Specifically, the coordinates are (*x* = 0.584, *y* = 0.391) for YPO_4_:5% Eu, (*x* = 0.648, *y* = 0.350) for YP_0.5_V_0.5_O_4_:5% Eu, and (*x* = 0.623, *y* = 0.372) for YVO_4_:5% Eu. These values are situated near the ideal red chromaticity coordinates of (*x* = 0.67, *y* = 0.33), confirming the samples exhibit strong red luminescence.^34^

## 4. Conclusion

YP_(1−*x*)_V_*x*_O_4_:5% Eu^3+^ was synthesized successfully using the combustion method at 900 °C. The XRD patterns of YP_(1−*x*)_V_*x*_O_4_:5% Eu^3+^ revealed a tetragonal crystal structure, whereas the SEM and TEM images illustrated uniformly spherical particles with a size of 20 nm. The aggregation of YP_(1−*x*)_V_*x*_O_4_:5% Eu^3+^ nanoparticles still occurred. The study on the optical properties of the obtained samples using fluorescence spectroscopy focused on how they change when phosphorus (P) is replaced by vanadium (V). The analysis leveraged Judd–Ofelt theory to calculate parameters such as intensity parameter, branching ratio, and quantum efficiency. Additionally, the CIE chromaticity coordinates were determined, revealing that the emission properties closely matched the standard red color, a desirable feature for applications like display technologies and lighting.

## Data availability

All data and materials generated or analyzed during this study are included in this published article and are available from the corresponding author upon reasonable request.

## Author contributions

Nguyen Vu: methodology, conceptualization, project administration, and writing – original draft; Ngo Khac Khong Minh: investigation and formal analysis; Thai Thi Dieu Hien: investigation and formal analysis; Pham Duc Roan: validation and supervision; Lam Thi Kieu Giang: investigation and data curation; Nguyen Thanh Huong: funding acquisition and investigation; Hoang Thi Khuyen: software and visualization; Pham Thi Lien: investigation and formal analysis; Dinh Manh Tien: investigation and formal analysis; Nguyen Trung Kien: software, data curation, and writing – review and editing; Dao Ngoc Nhiem: data curation and validation.

## Conflicts of interest

The authors declare no conflict of interest.
